# Development and Validation of a Short Version (PAIC6) of the Pain Assessment in Impaired Cognition Scale

**DOI:** 10.1002/ejp.4795

**Published:** 2025-02-08

**Authors:** Vivien Schreiber, Miriam Kunz, Wilco Achterberg, Jenny T. van der Steen, Frank Lobbezoo, Bernhard Langner, Stefan Lautenbacher

**Affiliations:** ^1^ Living Lab for Dementia Research (BamLiD) University of Bamberg Bamberg Germany; ^2^ Medical Faculty, Department of Medical Psychology and Sociology University of Augsburg Augsburg Germany; ^3^ Department of Public Health and Primary Care Leiden University Medical Center Leiden the Netherlands; ^4^ Radboud University Medical Center Department of Primary and Community Care and Radboudumc Alzheimer Center Nijmegen the Netherlands; ^5^ Department of Orofacial Pain and Dysfunction, Academic Centre for Dentistry Amsterdam (ACTA) University of Amsterdam and Vrije Universiteit Amsterdam Amsterdam the Netherlands; ^6^ Department of Orofacial Pain and Jaw Function Faculty of Odontology, Malmö University Malmo Sweden; ^7^ Retirement Home House Malta Malteser Hospital Berlin‐Charlottenburg Berlin Germany

**Keywords:** dementia, impaired cognition, PAIC15, pain assessment, short scale

## Abstract

**Background:**

Observer pain scales are commonly used to assess pain in individuals with impaired cognition. However, nursing staff have highlighted that extremely tight time schedules and increasing workload demands prevent regular use. With the development of a short version of the Pain Assessment in Impaired Cognition (PAIC15), we aimed to reduce implementation barriers in everyday clinical practice.

**Methods:**

We developed a new 6‐item short version (PAIC6) in a first sample (*N* = 59) and validated its psychometric properties in a second sample (*N* = 250) of older individuals with cognitive impairments. The item reduction and evaluation involved four steps. First, we used Sample 1 to exclude items based on item quality statistics (e.g., difficulty, reliability). Second, the Partial Credit Model (PCM) was utilised for further reduction using again Sample 1. Third, an expert panel evaluated the preceding steps and suggested a draft short version with six items (PAIC6). Fourth, psychometric properties of the short version were evaluated in the independent Sample 2. Thereafter, the final short version was approved.

**Results:**

The new PAIC6 showed a high correlation with the total scale PAIC15 (*r* = 0.870), good reliability (Cronbach's *α* = 0.684), and high convergent construct validity, as observed by a high correlation with the established Pain Assessment in Advanced Dementia (*r* = 0.602).

**Conclusions:**

Overall, we developed a valid, reliable, and clinically valuable PAIC6 that allows a more time‐efficient pain assessment, by reducing the assessment time from 5 min to approximately 2 min (60% time saving).

**Significance:**

Observer pain scales are commonly used to assess pain in individuals with impaired cognition. However, nursing staff have highlighted that extremely tight time schedules and increasing workload demands prevent regular use. To address this, we developed PAIC6, a short version of the Pain Assessment in Impaired Cognition 15 (PAIC15). PAIC6 includes six items and takes 2 min for completion after training, realising a 60%‐time reduction compared to the original scale while keeping the psychometric quality high.

## 1 Introduction

Observer assessment is the commonly used method for diagnosing and assessing pain in individuals with impaired cognition when self‐report starts to fail (Achterberg et al. 2013, 2020; Herr, Zwakhalen, and Swafford 2017). However, despite their clinical effectiveness in these persons (Chow et al. 2016; Hadjistavropoulos et al. 2014), time constrains are often highlighted by nurses as major barrier for implementation of observational pain assessment tools into daily routine (Burns and McIlfatrick 2015; Knopp‐Sihota, Dirk, and Rachor 2019; Minaya‐Freire et al. 2020).

The various observational pain assessment tools differ regarding the necessary observation time, that is, how long a patient should be observed, and the scoring time, that is, how long it takes to fill in the scores. Most tools take between 1 and 8 min for observation and 2–5 min for scoring (Felton et al. 2021; McGuire et al. 2016). These time differences are partly due to differences in item complexity, observation difficulty, rating systems (see, Chow et al. 2016; Lints‐Martindale et al. 2012) and most importantly due to the number of items included.

The observer scale ‘Pain Assessment in Impaired Cognition 15’ (PAIC15; Kunz et al. 2020) designed by a European initiative as an internationally agreed‐upon meta‐tool consists of 15 items found to be best possible amongst 12 established observer scales (Corbett et al. 2014). Therefore, its clinical applicability and handiness would also benefit from a scale shortening. Given that the PAIC15 scale was developed with a pragmatic focus (easy handling, simple items, clear content coverage) (Kunz et al. 2020), reducing the number of items could be dared without losing psychometric quality. In the present form, approximately 5 min are required for observation and scoring the PAIC15. Consequently, with a reduction to 5–10 items, we anticipated a time reduction from 5 to 2–3 min.

Successful shortening should still allow for covering all three key domains of pain assessment, namely “facial expression”, “body movements”, and “vocalisation” as recommended (Husebo et al. 2012; Lints‐Martindale et al. 2012; AGS: The Management of Chronic Pain in Older Persons 1998). Further, we aimed to reach a minimum item reduction of 50% in order to achieve noticeable timesaving.

We employed a four‐step approach for scale shortening and evaluation. Step 1 involved computing classical item statistics for each item as a pre‐selection of psychometric favourable items. In Step 2 we implemented the Partial Credit Model (PCM), only using the remaining items from Step 1. The PCM allows for identifying and removing items with low discriminative power, which are items that cannot effectively differentiate the latent variable (pain intensity) (Tennant and Conaghan 2007). In Step 3 we formed an expert panel with a multi‐professional background to evaluate the statistical item selection in Steps 1 and 2, mainly in terms of comprehensibility, user‐friendliness, and applicability of the remaining items in everyday care. In Step 4, we evaluated the draft short version in a second independent large sample to validate the psychometric quality of the draft short version and to end with a final short version.

## 2 Methods

The complete development of a short version out of PAIC 15 consisted of two parts: (1) analyses for item reduction were conducted to form a draft short version using the data of Sample 1, and (2) this draft short version was evaluated using the data of the independent Sample 2, to come up with a final short version.

### 2.1 Participants

*Sample 1* (*N* = 59 with 51 females; age: *M* = 87.10; SD = 7.89; range = 66–102) was recruited from a geriatric ward of a hospital and a nursing home in Berlin: Malteser‐hospital Berlin‐Charlottenburg and the nursing home Malta, respectively. Sample size was based on a posteriori power analysis (GPower) based on the Partial Credit Model and using the noncentral chi‐squared distribution while expecting large effect sizes, given that all items of the PAIC15 have already undergone critical item selection. This part of the study was initiated as a cooperative project between the Berlin institutions and the University of Bamberg. Data collection took place from April to August 2020 in Berlin. Most participants were tested in the morning. Participants received their usual medication before the observer assessment of pain. To determine the cognitive status as well as functional disability of the participants, the Global Deterioration Scale (GDS; Reisberg et al. 1982) and Barthel Index BI (Mahoney and Barthel 1965) were assessed, but outside of the pain assessment session. The GDS was used to assess the severity of dementia, while the BI assessed the ability to perform activities of daily living. Participant provided written informed consent prior to their participation. The study protocol was approved by the ethics committee at the Otto‐Friedrich University of Bamberg, as the study was conducted as a collaborative project between the hospital and nursing home in Berlin and the University of Bamberg.

*Sample 2* consisted of 250 participants (156 females; age: *M* = 85.33; SD = 7.50; range = 65–108). Participants were recruited in the Netherlands from several nursing homes' locked wards with 24/7 oversight. In these departments, most individuals have a clinical diagnosis of dementia. The GDS was used to assess the severity of dementia. Excluded were participants with a life expectancy shorter than 1 week. In Sample 2, a family member or legal representative in most of the cases, especially in those with severe dementia, provided written informed consent. In some cases, this was done together with the participant, when possible, while in other cases, the family member or representative provided consent on behalf of the participant alone. A more detailed description of the data collection process can be found elsewhere (van der Steen, Waal, and Achterberg 2021). This study protocol was approved by the Medical Ethics Review Committee of the Leiden University Medical Center.

### 2.2 Material and Protocol

#### 2.2.1 Protocol

##### 2.2.1.1 Sample 1

Two nurses conducted the observational pain assessment; applying the PAIC 15 first followed by another observational pain scale, namely the DoloPlus2 (Lefebvre‐Chapiro 2001) with a maximum total duration of 15 min per observation. The DoloPlus2 was the scale that had already been implemented for some time and that the nurses were familiar with from its regular use in everyday care. Although the nurses did not have much experience with the PAIC15, the e‐training (https://paic15.com/en/e‐training‐en/) made it possible to train the nurses quickly and reliably to keep the assessment quality consistently high for both scales^1^. Each participant was observed during rest (e.g., sitting in a chair while watching TV), and during mobilisation (e.g., transfer in bed and getting up, walking, and getting dressed in the morning). Given that pain is more likely to occur during mobilisation, we focused on the PAIC15 scores assessed during this condition for further analyses. Both nurses were simultaneously present and filled in the PAIC15 independently. The Rasch Partial Credit Model (PCM) favours scoring of a single person as a starting point because the model claims that responses are ordinal and reflect individual performance or ability. The combination of two raters can violate this assumption, as the computation of mean values generates artificial values. We ultimately chose to use Rater A's ratings. Upon reviewing the ratings, it became clear that Rater A provided more differential and graded assessments compared to Rater B. To further illustrate this, we first compared internal consistency and inter‐item correlations for both raters. The internal consistency was particularly high for Rater B (Cronbach's *α* = 0.932), which is often interpreted as redundancy or lower discriminatory ability of the items (Taber 2018). In contrast, Rater A showed well‐balanced ratings, with satisfactory internal consistency (Cronbach's *α* = 0.871), indicating a more differential and graded scoring of the items. When comparing the inter‐item correlations, Rater B showed especially high inter‐item correlations (mean Rater A = 0.38 and mean Rater B = 0.47, difference between raters: *t*(163) = −3.58, *p* < 0.001), indicating a stronger overlap and a lower differentiation ability of the ratings by Rater B. Thus, Rater B showed less differential and graded assessment, potentially missing finer behaviour distinctions.

##### 2.2.1.2 Global Deterioration Scale (GDS; Reisberg et al. 1982)

The GDS is used to assess cognitive functioning in patients with primary degenerative dementia. It divides dementia into seven severity levels from no cognitive decline (stage 1) to very severe cognitive decline (stage 7). The GDS has good test–retest reliability (Gottlieb, Gur, and Gur 1988) and a high external validity (Mavioglu et al. 2006; Moreno 2003; Reisberg et al. 1982). The GDS was assessed outside of the pain assessment session as part of a general cognitive assessment in everyday clinical care. In Sample 1 the GDS was filled out in German and in Sample 2 in Dutch.

##### 2.2.1.3 Barthel Index (BI; Mahoney and Barthel 1965)

The BI assesses functional impairments of geriatric populations to estimate their care needs in everyday life. It consists of 10 activities of daily living (ADL) that are scored with two points (0 or 5; e.g. bathing), three points (0, 5 or 10; e.g. toilet use) or four points (0, 5, 10 or 15; e.g. transfers). The final total score can reach a maximum of 100. The BI shows good reliability: Cronbach's *α* = 0.94 (dos Santos Barros et al. 2022). The Barthel index (sum score) was only assessed in Sample 1, conducted in German, prior to the pain assessment session.

##### 2.2.1.4 Sample 2

The observational pain assessments were conducted by physicians or trained staff members and started with the assessment of Pain Assessment in Advanced Dementia (PAINAD), followed by the evaluation by the PAIC15. The physicians could hand over the assessment to staff members if an introduction to the observations was given beforehand. In this sample, observational pain assessment was only conducted during resting conditions, given that the severe cognitive and functional limitations in this sample did not allow for mobilisation of all patients and pain became sufficiently detectable already in this resting condition.

#### 2.2.2 Observational Pain Assessment

##### 2.2.2.1 Pain Assessment in Impaired Cognition (PAIC15; Kunz et al. 2020) (Assessed in Sample 1 and 2)

The PAIC15 is a 15‐item observer scale administered by trained healthcare professionals to assess pain in patients with impaired cognition. It consists of three sub‐scales: facial expression, body movements, and vocalisation. The items (e.g., facial expression: “Frowning—lowering and drawing brows together”; body movements: “Freezing—stiffening, avoiding movement, holding breath” and vocalisation: “Shouting—using a loud voice to express words”) are recorded on a 4‐point scale. The raters were trained by successfully completing the 30‐min PAIC 15 e‐training (https://paic15.com/en/e‐training‐en/). In the training, items are explained by video examples, and PAIC15 scoring is practiced by training videos, including expert feedback. The PAIC15 shows very high inter‐rater reliability for all three scales (Cohen's kappa for facial expression: 0.91, vocalisation: 0.93, and body movement: 0.92; Kappesser et al. 2020). The PAIC15 has been translated into 11 languages. In Sample 1, the PAIC15 was used in German; in Sample 2, in Dutch.

##### 2.2.2.2 Pain Assessment in Advanced Dementia (PAINAD; Warden, Hurley, and Volicer 2003) (Assessed in Sample 2)

The PAINAD is a well‐established observer pain behaviour assessment and consists of five categories of potential pain behaviour: breathing, negative vocalisation, facial expression, body language, and controllability; rated in three categories from 0 (normal) to 2 (very noticeable). The PAINAD is recommended for pain assessment in individuals with dementia, as it covers the first three AGS domains (“facial expression,” “body movements,” and “vocalisation”), has high validity, especially in terms of agreement with self‐reported pain, and strong test‐rest reliability (Herr, Zwakhalen, and Swafford 2017; Lints‐Martindale et al. 2012). The PAINAD overall demonstrates good reliability: Cronbach's *α* = 0.80 (Fry and Elliott 2018). The total score can reach from 0 to 10. The PAINAD was applied in Sample 2 in Dutch and used to calculate the convergent construct validity (correlation of the new PAIC short version with an established pain indicator also assessed by the PAINAD).

### 2.3 Data Analysis

**FIGURE 1:**
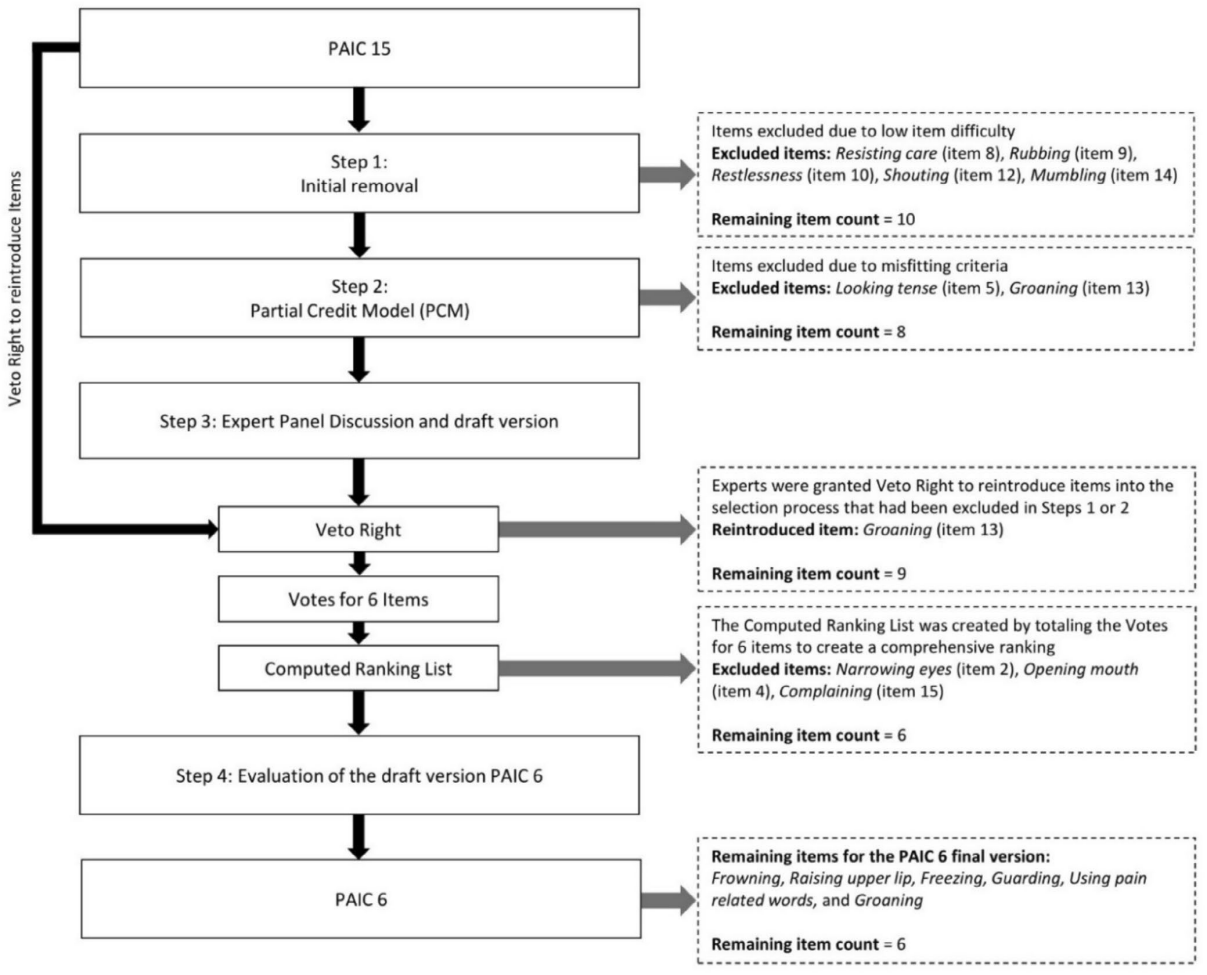
Overview of the steps used to develop the short version PAIC6 out of PAIC15. In Step 1 we used item statistics (psychometric analyses) to exclude items. In Step 2 we applied the Partial Credit Model (PCM) reduction approach for the remaining items. In Step 3 an Expert Panel had the task of reviewing the item selection. In Step 4 we evaluated the draft short version further. In the end, six items remained for the PAIC6 final version.

The data were analysed using descriptive and inferential statistics to evaluate the current item pool and to identify items eligible for a short version. Statistical analyses were performed using R version 4.2.2 (2022‐10‐31 ucrt) in RStudio version 2022.07.2 + 576, open‐sourced under an AGLP v3 licence. PCM analysis was performed using the eRm R package (Mair, Hatzinger, and Maier 2021; Mair and Hatzinger 2007). The significance criterion alpha for all tests was 0.05. For Sample 1, the PAIC15 during mobilisation was used, as this dataset had an optimal distribution of item scores for scale shortening. In Sample 2, the participants were more severely impaired, allowing only for assessment of the PAIC15 at rest. Already in the rest condition, pain reached levels, which were sufficient to observe and score non‐verbal pain responses in sample 2. The item reduction to a draft short version and the evaluation of this draft short version to finalise the short scale took place in four steps (see also Figure 1).

#### 2.3.1 Step 1: Initial Removal

We started in Step 1 as a pre‐selection of psychometric favourable items with an examination of the original 15 items of PAIC15 using several established tests highlighting items with psychometric high quality. Thus, this step was based on item statistics that are described as useful criteria for item reduction (Goetz et al. 2013): item difficulty, item correlation with the total score, scale reliability when an item is excluded, and missing values. Item difficulty is measured from 0 to 1, where high values indicate that an item was frequently scored (“easy”) whereas low values indicate infrequent scoring (“difficult”; Sim and Rasiah 2006). Based on the PAIC15 development approach (Kunz et al. 2020), items with difficulties < 0.10 or > 0.90 were considered too easy or too difficult and were therefore excluded. Item representativeness for the total PAIC15 score was evaluated by the correlation of every item score with the total score (item‐total correlation). The established criterion for excluding items was *r* < 0.4 (Erhart et al. 2010). Next, we considered how the reliability of the total scale changed when individual items were excluded. Cronbach's α was used, with values ≥ 0.70 being considered an indication of good internal consistency (Bland and Altman 1997; DeVellis and Thorpe 2022). We excluded items that lowered the scale's reliability. Items with more than 50% missing values were excluded, as in this case difficulties in observing the associated behaviour and in completing the evaluation can be assumed. The criteria were considered one after the other, excluding all items that met at least one of these exclusion criteria.

#### 2.3.2 Step 2: Partial Credit Model

The Partial Credit Model (PCM; Masters 1982) was used as an additional finer filtering process following the preselection of items in Step 1. As a generalisation of the dichotomous Rasch model, the PCM offers an interpretation‐free exclusion method based on fixed criteria for purely statistical scale reduction, a method successfully implemented in previous studies (see Cantó‐Cerdán et al. 2021; Hamilton et al. 2021; van Nispen tot Pannerden et al. 2009).

The PCM allows consideration of underlying measurement constructs as it provides an estimate of latent variables by establishing a model across all individuals based on the given item responses. The latent variable was in our case the intensity of pain experience. The model thus makes it possible to identify items with too low discriminative power (Tennant and Conaghan 2007). The exclusion of items by the PCM had one restriction, namely that each of the three key domains of pain behaviours (“facial expression,” “body movements,” and “vocalisation”) was still represented with at least two items.

To interpret the PCM model fit, we analysed commonly used PCM statistics (Pesudovs et al. 2007; Smith, McCarthy, and Anderson 2000; Tennant and Conaghan 2007). The item that was excluded first met the most misfit criteria during iteration:

1. Item‐trait interaction: The chi‐square statistic evaluates the fit between the expected and observed item structure, with a non‐significant *p*‐value (*α* > 0.05) indicating a satisfactory model fit (Hamilton et al. 2021).
2. Item infit and outfit mean square error term (MSQ): We calculated the MSQ for an item to determine whether an item corresponds to the linear function. Infit and outfit mean squares between 0.70 and 1.30 indicate a good model fit (Pesudovs et al. 2007). While values below 0.70 indicate possible item redundancy, values above 1.30 indicate a deviation from the measurement construct of the overall scale (Pesudovs et al. 2007).
3. Standardised infit static: Items with a highly negative standardised infit static (less than −2) were excluded since item redundancy must be assumed in this case (Beaton, Wright, and Katz 2005).
4. Separation index: The separation index was calculated, which serves as a reliability descriptor. Acceptable values, indicating good reliability, are > 0.80 for person separation reliability and > 0.90 for item separation reliability (Cantó‐Cerdán et al. 2021). Further details on the calculation and interpretation basis can be found in Mair and Hatzinger (2007).

#### 2.3.3 Step 3: Expert Panel Discussion and Draft Short Version

An expert panel was formed to select the best possible items in terms of comprehensibility, user‐friendliness, and applicability (see Figure 1) based on the statistical pre‐selection in step 1 and 2. Six experts from a multi‐professional background all dealing with care, management, or research in aged individuals or persons with dementia participated: physician (*n* = 1), dentist (*n* = 1), geriatric nurses (*n* = 2), and psychologists (*n* = 2). The experts were given a veto right to indicate whether an item excluded during step 1 and 2 was indispensable. In this case, a consensus discussion was started until general agreement and a possible re‐introduction of the item. Thereafter, we asked the experts to vote for those six items amongst the remaining items after step 1 and 2 they wanted to be included in a shortened PAIC. That the experts should vote for six items was based on our aim that the PAIC short version should comprise not more than six items to achieve considerable time reduction. We summed up the votes for each item and computed a ranking list. The six items ranking highest were selected for the draft short PAIC version.

#### 2.3.4 Step 4: Evaluation of the Draft Short Version

For the evaluation of the draft short version, we used an independent Sample 2 as methodological guidelines recommend (Goetz et al. 2013; Hinkin 1998; Smith, McCarthy, and Anderson 2000). We computed the correlation of the draft short version with the PAIC15 (scale‐total correlation) and the internal consistency using Cronbach's *α*. To examine convergent construct validity, we calculated the correlation of the draft short version with the Pain Assessment in Advanced Dementia scale (PAINAD; Warden, Hurley, and Volicer 2003).

## 3 Results

Descriptive characteristics of both samples are displayed in Table 1. Both samples are comparable with regard to age. However, the degree of cognitive impairment (GDS) was more pronounced in the Dutch Sample 2 than in the German Sample 1. Functional impairments amongst participants were assessed only in Sample 1 using the Barthel Index. Most participants reached moderate to severe dependency scores. PAIC15 scores were comparable between samples. Given that a PAIC score of ≥ 3 has been suggested to indicate pain (van der Steen, Waal, and Achterberg 2021), average PAIC15 scores indicated the presence of pain in both samples.

**TABLE 1:** Descriptive characteristics of Sample 1 and Sample 2.

	Sample 1		Sample 2	
*N*	59		250	
Age in years (mean (SD))	87.10	(7.89)	85.33	(7.50)
Sex (females/males)	51/8		156/94	
Cognitive decline, GDS (*N* (%))^a^				
None (level 1)	14	(8)	0	(0)
Very mild (level 2)	17	(10)	1	(0)
Mild (level 3)	9	(5)	4	(2)
Moderate (level 4)	9	(5)	22	(9)
Moderate severe (level 5)	7	(4)	67	(27)
Severe (level 6)	3	(2)	117	(47)
Very severe (level 7)	0	(0)	39	(16)
Functional dependency, BI (*N* (%))^b^				
None—mild (BI: 91–100)	5	(8)	—	
Moderate—severe (BI: 21–90)	51	(86)	—	
Total dependency (BI: 0–20)	3	(5)	—	
PAIC15 (mean (SD))^c^	4.85	(4.78)	4.53	(5.00)

^a^ GDS (Global Deterioration Scale).

^b^ BI (Barthel Index).

^c^ PAIC15 (Pain Assessment in Impaired Cognition Scale). – indicates that BI was not observed in Sample 2.

### 3.1 Step 1: Initial Removal

Table 2 shows the results of the item analysis which served as first selection to find psychometric favourable items. We excluded five items due to an item difficulty below 0.10: *Resisting care* (item 8), *Rubbing* (item 9), *Restlessness* (item 10), *Shouting* (item 12), and *Mumbling* (item 14); that is, these items were scored very infrequently. All remaining 10 items showed acceptable item‐total correlations between 0.45 and 0.76. No item was excluded when considering the reliability when the item was dropped, that is, no item would have increased the overall scale reliability (Cronbach's *α*) by its exclusion. Only *Mumbling* (item 14) had missing values, although these are negligible at 1.69%. Thus, after Step 1 10 items remained (see Figure 1): All five items of the facial expression scale remained, as well as Freezing (item 6) and Guarding 7 (item 7) of the body movements scale and Using pain‐related words (item 11), Groaning (item 13), and Complaining (item 15) of the vocalisation scale. The internal consistency of the remaining 10 items was high (Cronbach's *α* = 0.888).

**TABLE 2:** Item Analysis of all 15 items of the Pain Assessment in Impaired Cognition (PAIC 15) scale (Sample 1).

PAIC items		Missings	Mean	SD	Item difficulty^a^	Item‐total correlation^b^	*α* if deleted^c^
Facial Expression							
1	Frowning	0.00%	0.51	0.68	0.25	0.73	0.85
2	Narrowing eyes	0.00%	0.46	0.73	0.23	0.68	0.86
3	Raising upper lip	0.00%	0.32	0.51	0.16	0.64	0.86
4	Opening mouth	0.00%	0.93	0.64	0.47	0.58	0.86
5	Looking tense	0.00%	0.78	0.79	0.39	0.76	0.85
Body Movements							
6	Freezing	0.00%	0.44	0.60	0.22	0.74	0.85
7	Guarding	0.00%	0.44	0.68	0.22	0.70	0.86
8	Resisting care	0.00%	0.00	0.00	—	—	0.88
9	Rubbing	0.00%	0.02	0.13	0.02	0.46	0.87
10	Restlessness	0.00%	0.02	0.13	0.02	0.46	0.87
Vocalisation							
11	Using pain‐related words	0.00%	0.32	0.65	0.16	0.74	0.85
12	Shouting	0.00%	0.00	0.00	—	—	0.88
13	Groaning	0.00%	0.32	0.65	0.11	0.45	0.87
14	Mumbling	1.69%	0.09	0.28	0.09	0.39	0.87
15	Complaining	0.00%	0.20	0.48	0.10	0.76	0.85

*Note:* Sample 1 was used. Total scale Cronbach's *α* = 0.871.

^a^ Refers to the probability of an item being rated high or low.

^b^ Item‐total correlation corrected for item overlap and scale reliability.

^c^ Cronbach's *α* if an item is deleted. — indicates item score > 1 was not observed, therefore item difficulty and item‐total correlation could not be calculated. Excluded items are highlighted in grey.

### 3.2 Step 2: Partial Credit Model

The Partial Credit Model (PCM; Masters 1982) was used as an additional finer filtering process following the preselection of items in Step 1. Table 3 shows the fit statistics for three iterations of the PCM that we calculated. It is important to note that further iterations would computationally have been possible. However, we set the predefined criteria to stop with further iterations when each of the three key domains of pain behaviours (“facial expression,” “body movements,” and “vocalisation”) was no longer represented by at least two items. We excluded one misfitting item after each iteration.

**TABLE 3:** Partial Credit Model (PCM): Iterations 1 to 3 (Sample 1).

PAIC items		*Chisq*	DF	*p*	outfit MSQ	infit MSQ	outfit *Z*	infit *Z*
Iteration 1								
Facial expression								
1	Frowning	50.50	50	0.454	0.99	0.89	0.06	−0.48
2	Narrowing eyes	40.53	50	0.828	0.79	0.96	−0.38	−0.09
3	Raising upper lip	37.14	50	0.911	0.73	0.83	−0.75	−0.80
4	Opening mouth	52.99	50	0.360	1.04	1.01	0.26	0.12
5	Looking tense	27.02	50	0.997	0.53	0.61	−2.43	−2.36
Body movements								
6	Freezing	35.93	50	0.933	0.70	0.81	−1.13	−0.98
7	Guarding	45.48	50	0.655	0.89	1.05	−0.19	0.32
Vocalisation								
11	Using pain related words	30.76	50	0.985	0.60	1.06	−0.50	0.29
13	Groaning	93.65	50	< 0.001	1.84	1.51	1.46	1.67
15	Complaining	22.49	50	1.000	0.44	0.70	−0.80	−1.03
Iteration 2								
Facial Expression								
1	Frowning	51.78	50	0.404	1.01	0.90	0.14	−0.43
2	Narrowing eyes	42.71	50	0.758	0.84	0.99	−0.30	0.05
3	Raising upper lip	38.04	50	0.892	0.75	0.85	−0.78	−0.71
4	Opening mouth	44.37	50	0.698	0.87	0.87	−0.64	−0.71
Body movements								
6	Freezing	33.48	50	0.965	0.66	0.77	−1.49	−1.21
7	Guarding	41.18	50	0.808	0.81	0.96	−0.51	−0.09
Vocalisation								
11	Using pain related words	24.29	50	0.999	0.48	0.91	−0.89	−0.25
13	Groaning	77.43	50	0.008	1.52	1.46	1.09	1.53
15	Complaining	19.14	50	1.000	0.38	0.65	−1.13	−1.27
Iteration 3								
Facial expression								
1	Frowning	50.19	49	0.426	1.00	0.91	0.10	−0.41
2	Narrowing eyes	47.22	49	0.545	0.94	1.07	−0.00	0.38
3	Raising upper lip	40.47	49	0.802	0.81	0.93	−0.55	−0.29
4	Opening mouth	49.62	49	0.448	0.99	0.96	0.04	−0.13
Body movements								
6	Freezing	32.42	49	0.967	0.65	0.76	−1.56	−1.25
7	Guarding	38.55	49	0.858	0.77	0.95	−0.63	−0.16
Vocalisation								
11	Using pain related words	27.82	49	0.994	0.56	0.94	−0.69	−0.12
15	Complaining	19.37	49	1.000	0.39	0.62	−1.10	−1.38

*Note:* Sample 1 was used. Items with a significant chi‐square *p*‐value were excluded. Items with an infit or outfit MSQ outside the range of 0.70–1.30 were excluded. Items with a highly negative standardised infit static (*z*‐value > −2) were excluded. Iteration 3: Item 15 was retained despite misfitting criteria to meet our criterion of representing three of the pain assessment domains recommended by the American Geriatrics Society: “facial expression,” “body movements,” and “vocalisation,” each with at least two corresponding items. The separation index for the remaining eight items after the PCM was high (person separation reliability > 0.99 and item separation reliability > 0.99), indicating high reliability. Excluded items are highlighted in grey.

The initial 10 items had outfit mean square values between 0.44 and 1.84 and infit mean square values between 0.61 and 1.51, where infit and outfit mean squares between 0.70 and 1.30 indicate a good model fit. *Z*‐standardised values ranged for infit mean square between −2.36 and 1.67, where highly negative standardised infit static (> −2) indicates item redundancy (Beaton, Wright, and Katz 2005). First, after iteration 1, Looking Tense (item 5) was excluded because it met most misfitting criteria (outfit and infit MSQ < 0.70 and infit *Z* < −2). Second, after iteration 2, we removed Groaning (item 13; chi‐square *p* < 0.05 and outfit MSQ > 1.30). Third, after iteration 3, Complaining (item 15) exhibited a lower‐than‐desired MSQ (outfit = 0.39 and infit = 0.62), which indicates the possibility of item redundancy (Linacre 2002). In addition, Freezing (item 6) and using pain related words (item 11) showed a slightly lower‐than desired outfit MSQ (item 6 outfit MSQ = 0.65 and item 11 outfit MSQ = 0.56). Considering that item redundancy can never be completely avoided, we opted to keep item 15 as well as items 6 and 11 to meet our criterion of representing the three AGS‐recommended pain assessment domains (“facial expression,” “body movements,” and “vocalisation”) with at least two corresponding items. The calculation of PCM was thus completed. The item selection after Step 2 reduced the item pool by two items and left eight items over (see Figure 1): Frowning (item 1), Narrowing eyes (item 2), Raising upper lip (item 3), and Opening mouth (item 4) of the facial expression scale, as well as Freezing (item 6) and Guarding (item 7) of the body movements scale, and using pain‐related words (item 11) and Complaining (item 15) of the vocalisation scale. The internal consistency of the remaining eight items was high (Cronbach's *α* = 0.868).

### 3.3 Step 3: Expert Panel Discussion and Draft Short Version

The expert panel had the task of reviewing the selection process and the remaining items. The experts could veto and reintroduce items, which were already excluded in Step 1 or Step 2 (see Figure 1). The experts reintroduced the item Groaning (item 13) which had been excluded in Step 2 due to misfitting criteria in the PCM that indicated a deviation from the measurement construct of the overall scale and item redundancy. The experts justified their decision by stating that Groaning is an essential component of pain response in the context of cognitive impairment. With the reintroduction of Groaning (item 13), nine items remained: Frowning (item 1), Narrowing eyes (item 2), Raising upper lip (item 3), Opening mouth (item 4), Freezing (item 6), Guarding (item 7), Using pain‐related words (item 11), Groaning (item 13) and Complaining (item 15). Now, each expert was asked to vote for six items out of the nine that they deemed indispensable for a shortened version. All six experts unanimously voted for including Freezing (item 6) and using pain‐related words (item 11) in the short version (100% expert voting). Frowning (item 1) received five expert votes (83% expert voting) while Raising upper lip (item 3), Guarding (item 7), and Groaning (item 13) each received four expert votes (66% expert voting). Opening mouth (item 4) received two expert votes (33% expert voting). Narrowing eyes (item 2) received one expert vote (17% expert voting). Finally, Complaining (item 15) did not receive any expert votes (0% expert voting). Finally, we totaled the expert votes for each item to create a ranking list and find the six highest ranked items: Using pain‐related words (item 11) and Freezing (item 6) (6 votes), Frowning (item 1) (5 votes), Raising upper lip (item 3), Guarding (item 7) and Groaning (item 13) (all 4 votes). With selection of these six items for the draft short version, our criteria of including all three key domains of pain assessment (“facial expression,” “body movements,” and “vocalisation”) and reducing the item number by at least 50% were met. An overview of the item selection is also given in Table 4.

**TABLE 4:** Excluded items and reasons for item exclusion in the four steps.

PAIC items		Step 1 Initial removal	Step 2 Partial credit model	Step 3 Expert panel discussion		
				Veto right^a^	Votes for six items^b^	Computed ranking list^c^
Facial Expression						
1	Frowning	Retained	Retained		5 expert votes	Retained
2	Narrowing eyes	Retained	Retained		1 expert votes	
3	Raising upper lip	Retained	Retained		4 expert votes	Retained
4	Opening mouth	Retained	Retained		2 expert votes	
5	Looking tense	Retained	Excluded due to misfitting criteria (outfit and intfit MSQ < 0.70 and infit *Z* < −2)			
Body Movements						
6	Freezing	Retained	Retained		6 expert votes	Retained
7	Guarding	Retained	Retained		4 expert votes	Retained
8	Resisting care	Excluded due to low item difficulty < 0.01	—			
9	Rubbing	Excluded due to low item difficulty = 0.02	—			
10	Restlessness	Excluded due to low item difficulty = 0.02	—			
Vocalisation						
11	Using pain related words	Retained	Retained		6 expert votes	Retained
12	Shouting	Excluded due to low item difficulty < 0.01	—			
13	Groaning	Retained	Excluded due to misfitting criteria (chi‐square *p* < 0.05 and outfit MSQ > 1.30)	Retained	4 expert votes	Retained
14	Mumbling	Excluded due to low item difficulty = 0.09	—			
15	Complaining	Retained	Retained		0 expert votes	

*Note:* — indicates that an item was already excluded in Step 1 and was not considered further in Step 2.

^a^ Experts were granted Veto Right to reintroduce items into the selection process that had been excluded in Steps 1 or 2.

^b^ Experts were asked to vote for 6 items that they deemed indispensable for a shortened version.

^c^ The Computed Ranking List was created by totaling the expert points per item to create a comprehensive ranking list. Excluded items are highlighted in grey. The draft short version PAIC6 thus includes the items: Frowning, Raising upper lip, Freezing, Guarding, Using pain related words, and Groaning.

### 3.4 Step 4: Evaluation of the Draft Short Version

The draft version of PAIC6 was evaluated using an independent sample (Sample 2). Sample 2 included a wide range of dementia severities, was unique because of its high number and was—as a multi‐center study—representative for senior home residents in the Netherlands. However, we would have run into major problems if the reliability and other psychometric properties of the PAIC6 deteriorated in Sample 2 along with the severity of the disease. To put this concern to test, we divided Sample 2 into two data sets—2.i with GDS scores less than 6 (early‐stage dementia; *n* = 94) and 2.ii with GDS scores greater and equal to 6 (advanced dementia; *n* = 156), likely including non‐verbal individuals. For both sub‐samples, we were able to demonstrate a high internal consistency (Cronbach's *α*: 2.i = 0.72 and 2.i = 0.82). These reliability values are comparable to Sample 1 with a Cronbach's *α* = 0.87. In summary, these data suggests that both subsamples show a similar, very good, internal consistency—indicating the independence of the scale quality in this aspect from the level of dementia deterioration.

The mean PAIC6 score of Sample 2 was 1.14 (SD = 1.80; range = 0–13). The correlation of the draft short version PAIC6 with the PAIC15 total score was high (*r*(248) = 0.830, *p* < 0.001). As a measure of convergent construct validity, we found a high correlation between the draft short version PAIC6 and PAINAD (*r*(248) = 0.602, *p* < 0.001), which is only slightly lower than the correlation between PAIC15 and PAINAD (*r*(248) = 0.763, *p* < 0.001). Only item 13 had missing data of 10%. A large proportion of the items were rated with 0; ranging from 61% (item 1) to 94% (item 7). The draft short version PAIC6 has good reliability (Cronbach's *α* = 0.684).

**FIGURE 2:**
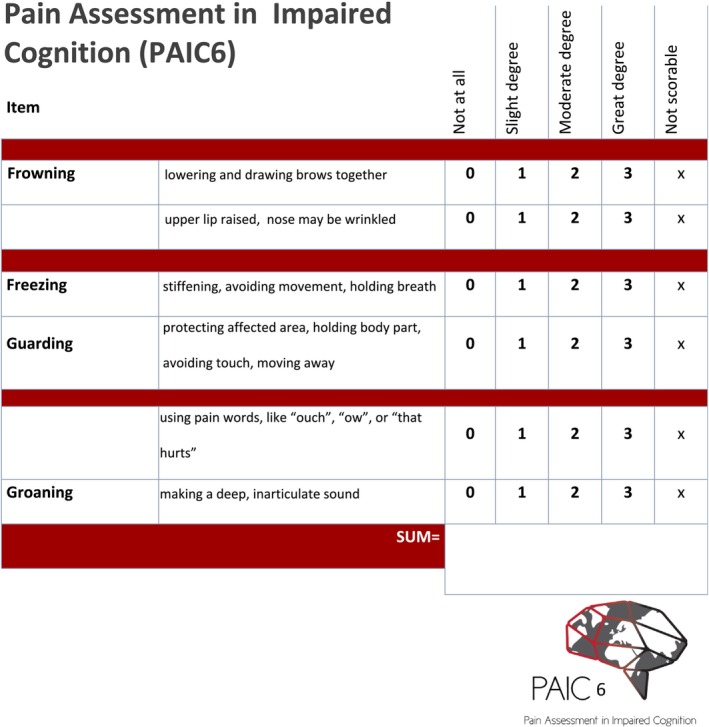
Form for the newly developed PAIC6, using the same layout as for the long version PAIC15 to help former users to quickly become familiar with the new scale.

After this final evaluation of the draft short version of PAIC6, we decided to maintain this version. Our final PAIC6 can be found in Figure 2 and includes the following items: Frowning and Raising upper lip of the facial expression scale, Freezing and Guarding of the body movements scale as well as using pain‐related words and Groaning of the vocalisation scale. This results in a final PAIC6 with a maximum total score of 18, where higher values indicate a higher level of pain.

### 3.5 PAIC6 Cut‐Off Values

We aimed to establish a cut‐off score for the PAIC6 as has been done for the PAIC15 to indicate when pain is like present. For the PAIC15 a total score of ≥ 4 has been suggested to indicate “possible pain”, while a total score of ≥ 5 has been suggested to indicate “pain” (van der Steen, Waal, and Achterberg 2021). Accordingly, we tried to establish a cut‐off score for the PAIC6. For Sample 2, we observed that 51% of participants were classified as experiencing pain for a PAIC6 total scores > 1, aligning with meta‐analyses that report pain frequencies of 46%–56% amongst individuals with dementia (Tan et al. 2015; van Kooten et al. 2016). Therefore, we suggest that a PAIC6 total score ≥ 1 indicate “possible pain”, while a total score of ≥ 2 indicate “pain.”

## 4 Discussion

Implementing psychometrically excellent observational pain assessment tools into nursing practice in senior homes is invaluable, given that pain assessment based on self‐report becomes more invalid when cognitive impairment accompanies increasing age (Achterberg et al. 2020; Chow et al. 2016; Lukas et al. 2013). However, enormous time pressure and immense workload of nursing staff, often make observational pain assessments challenging. Shortening available observational pain assessment tools by a few minutes might be critical for more successful implementation. With this study, we aimed to create a short version of the PAIC15 that decreases application time while maintaining its psychometric quality. With our 6‐item short version of the PAIC15 (called PAIC6), we achieved a total assessment time reduction from five to approximately 2 min (60% time saving). Furthermore, two major quality criteria were met: (i) the PAIC6 still covers the three main non‐verbal domains of pain recommended by the American Geriatric Society (AGS: The Management of Chronic Pain in Older Persons 1998) with two items for “facial expression,” “body movements,” and “vocalisation” and (ii) item count was reduced by at least half.

Maintaining the psychometric quality of the long version is particularly important when creating a short scale. The PAIC15 was initially created not to be the shortest scale possible but rather to ensure extensive coverage of the essential pain dimensions. For that purpose, the best items out of 12 established observational pain scales were selected to create a meta‐tool (Corbett et al. 2014; Kunz et al. 2020). Only after this meta‐tool had been developed, it was possible to reduce the item count further, creating the short version PAIC6. A high correlation between PAIC15 and PAIC6 total scores was observed, thus, suggesting high agreement between the long and short versions. Moreover, the PAIC6 demonstrated high convergent construct validity as observed by a high correlation with the PAINAD. PAINAD is considered a standard for observational pain assessment in individuals with dementia (Herr, Zwakhalen, and Swafford 2017; Lints‐Martindale et al. 2012), with high validity and reliability and significant importance in clinical application (Warden, Hurley, and Volicer 2003). Thus, the high agreement between PAIC6 and PAINAD speaks for a high validity of the PAIC6.

We placed a special focus on increasing time efficiency when creating a comprehensive pain screening tool that is applicable for routine use in daily clinical practice. There have been similar attempts to develop a short scale like ours. Van Nispen tot Pannerden et al. (2009) already demonstrated that by shortening the Pain Assessment Checklist for Seniors with Limited Ability to Communicate (PACSLAC) from 60 to 31 items, it was possible to reduce the scoring time by half. Moreover, attempts have been made to shorten the DoloPlus‐2; resulting in the Doloshort (Pautex et al. 2007) and the ALGOPLUS (Ratl et al. 2011). Although the Doloshort shows promising psychometric properties (Pautex et al. 2007, 2009), it lacks the inclusion of items referring to the facial expression. The ALGOPLUS (Ratl et al. 2011) was developed specifically as an acute pain assessment tool and is therefore, not applicable to all care situations. Furthermore, neither Doloshort nor ALGOPLUS have yet been included in major guidelines for the assessment of pain in older people, such as the German (Sirsch et al. 2012) or the UK (Schofield 2018) National Guidelines. Thus, several research groups have attempted to shorten observational scales to assess pain in dementia although the already existing scales are not outstandingly long. Doloplus‐2 (Lefebvre‐Chapiro 2001), Mahoney Pain Scale (MPS; Mahoney and Peters 2008), Mobilisation‐Observation‐Behaviour‐Intensity‐Dementia‐2 (MOBID‐2; Husebo et al. 2010), and PAINAD, include between 3 and 10 items and usually take 1–5 min to complete (Herr, Zwakhalen, and Swafford 2017). The PAIC with 15 items requires about 5 min to complete: consisting of 3 min of observation time and 2 min of scoring time. We assumed that the PAIC items are homogenous and do not differ in their item complexity (i.e., item length), observation difficulty (e.g., motor gestures might be easier to observe than facial expressions), and rating system (e.g., binary ratings or categorical ratings). For note, most PAIC6 items describe respondent (reflex‐like) behaviour, which is unlikely to vary substantially over time. Therefore, no item of the PAIC15 should be an outlier in application time. With this assumption, we anticipate a maximum time reduction from 5 min (300 s) with 15 items to 1.7 min (100 s) with five items; and consequently, a time reduction of approximately 2 min (120 s).

This accumulation of attempts to develop time efficient observational tools raises the question whether these attempts really meet the needs of the end‐users, namely the nursing staff. We would argue “yes”, given that prior studies underline that time constraints or “time required for application” are common barriers that hinder nurses to use observational pain assessment tools (Burns and McIlfatrick 2015; Knopp‐Sihota, Dirk, and Rachor 2019; Minaya‐Freire et al. 2020). One of the present authors (BL), a nurse and nurse supervisor, confirms that caregivers often refrain from using pain assessment scales and prioritise their intuitive expertise, due to a lack of. One difficulty might be that miniaturised tools—even if of best psychometric quality as we tried to develop—may raise scepticism and might be viewed as too concise to be good. Zwakhalen et al. (2006) pointed out this type of scepticism, which must be considered in the phase of implementation. For sure, there are other implementation barriers such as poor workplace conditions (Achterberg et al. 2020), and uncertainty regarding usage (Jonsdottir and Gunnarsson 2021) and interpretation of the scales (Jonsdottir and Gunnarsson 2021; Zwakhalen et al., 2018). However, the lack of time seems to be amongst these most pressing ones.

For the present development of the PAIC6, it was unavoidable that participants first filled in PAIC15, from which the six items of the PAIC6 were extracted. Follow‐up studies should also directly compare the new complete PAIC6 with the complete PAIC15. Future studies should also validate the PAIC6 for other situational contexts, besides the nursing home context, such as different hospital settings and hospice care. The varying clinical situations encompass variations in patients' mobility, dementia severity, and patient's ability to communicate and interact. Furthermore, investigating the PAIC6 in different subgroups with other forms of cognitive impairment, like individuals with intellectual disabilities, Huntington's disease Korsakoff's syndrome or Parkinson's disease, is conceivable. Previous studies using the PAIC15 have already shown good applicability in some of these subgroups (Defrin et al. 2021; Kunz et al. 2020; Oudman et al. 2023).

For persons, who are already using observational pain assessment tools, other than the PAIC6, the question may arise why they should change the diagnostic instrument. The PAIC15, and its short version PAIC6, offer several advantages: The PAIC15 was developed as a meta‐tool that integrates the best items from multiple well‐established pain assessment tools, by an international team and is translated into 11 languages. It also offers comprehensive e‐training modules for practicing its use efficiently (https://paic15.com/). All advantages of the PAIC15 also apply to the PAIC6.

However, despite these advantages, it is of course better to use any of the established observational scales for pain assessment in dementia instead of using none.

### 4.1 Limitations

There are limitations to be mentioned. The Dutch study (Sample 2) assessed the PAIC6 items during rest condition compared to the German study (Sample 1), which used observations during mobilisation. We did that for the following reason. Sample 1 participants were non‐frail and required mobilisation to recognisably display pain, while the frail individuals in Sample 2 showed sufficient pain behaviours already at rest. Inspection of the data showed that the two samples neither differed regarding the number of missing data nor regarding the distribution of the items scores. Observational scale developments have often started with non‐frail and only slightly cognitively impaired persons because the comparison with the subjective report of pain is of interest. Furthermore, participants were predominantly female. This uneven sex distribution can mainly be attributed to the high prevalence of women living in senior residences. It requires additional studies with a higher percentage of men, exploring the impact of sex on the psychometric qualities and applicability of the PAIC6. Additionally, the studies differed in their construct validation methods: DoloPlus2 was used for study 1, while PAINAD was used for study 2. However, both tools are widely recommended (McLennan et al. 2024), and as a benefit, we were able to obtain independent information about the construct validity, which corroborated each other.

### 4.2 Conclusion

We developed a valid, reliable, and clinically valuable short form of the PAIC15, namely the PAIC6, which requires only 2 min for completion after training, realising a 60%‐time reduction compared to the original PAIC15 scale. Thus, this shortened version of a worldwide available observer scale for assessing pain in persons with cognitive impairments considers the significant workload demands in the daily nursing practice, offering time‐saving benefits while keeping the psychometric quality high. Future studies should explore the application of PAIC6 in various subgroups (e.g., patients with developmental disabilities, Parkinson's disease) and in other contexts (e.g., hospital settings with post‐operative acute care or outpatient care).

## Author Contributions

This study was designed by S.L., M.K. and B.L. The experiment was performed by B.L., J.T.vdS. and W.A. The data was analysed by V.S., and the results were critically examined by all authors. V.S. and S.L had a primary role in preparing the manuscript, which was edited by M.K., W.A., J.T.vdS, F.L. and B.L. All authors have approved the final version of the manuscript and agree to be accountable for all aspects of the work.

## Conflicts of Interest

The authors delcare no conflicts of interest.

## Use of Artificial Intelligence

We did not use artificial intelligence (AI) or AI‐assisted technologies in the preparation of this manuscript.

## Endnotes

^1^
The DoloPlus2 assessed in study 1 was here not in the center of interest as validation tool. However, it might be informative to know the correlation between PAIC6 and DoloPlus2, which is (*r*(57) = 0.732, *p* < 0.001), which suggests a good construct reliability. This finding agrees with the result reported later in the text for the PAIC6 and PAINAD.
